# Supplementary material for: Pre-Clinical Evaluation of the Proteasome Inhibitor Ixazomib against Bortezomib-Resistant Leukemia Cells and Primary Acute Leukemia Cells
Source: Cells. 2021 Mar 17;10(3):665. doi: 10.3390/cells10030665 (PMC8002577; doi:10.3390/cells10030665)
Supplement: Supplementary file 1 [file cells-10-00665-s001.pdf]

Supplementary Materials

FIGURE S1: IXA vs BTZ inhibition profile of proteasome subunit activity in CEM (ALL) cells and their low/high BTZ-resistant sublines. β5, β5i, β1, and β1i-associated catalytic activity in cell extracts of CEM/WT, CEM/BTZ7 (low level BTZ resistance), and CEM/BTZ/BTZ200 (high level BTZ resistance) was assessed in the absence (control) or presence of increasing concentrations IXA or BTZ. Results depicted represent the absolute mean of the slopes ± SD of three separate experiments.

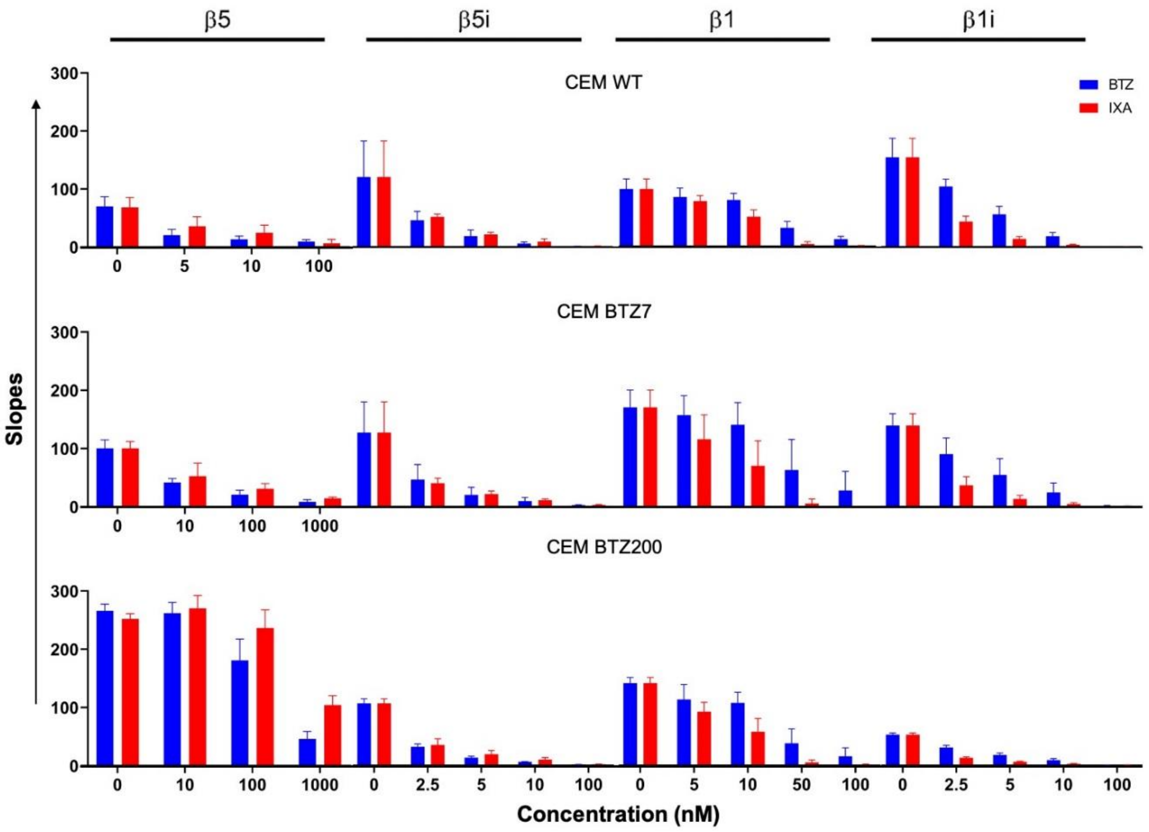

TABLE S1: Patients' characteristics

		ALL	AML
**Age, yrs mean ± SD**		5.8 ± 4.4	5.7 ± 5.5
**Female**		8	4
**Male**		7	5
**Blast %**		94 (range 86-98)	79 (range 30-93)
**Subtype**	**B-cell**	8	
	**T-cell**	4	
	**Unknown**	3	1
	**FAB M1**		1
	**FAB M2**		3
	**FAB M3**		-
	**FAB M4**		-
	**FAB M5**		4
	**FAB M6**		-

Table S2. Growth inhibitory effects of single drug IXA, DEX, and Ara-C and their concentration ranges used for the combination experiments.

	IC50 IXA	Concentration range IXA	IC50 DEX	Concentration range DEX	IC50 Ara-C	Concentration range Ara-C
T-ALL cells						
CEM/WT	0.018 ± 0.0	3.05E-05 – 0.0625	0.016 ± 0.0	3.05E-05 – 0.0625	-	-
CEM/BTZ7	0.115 ± 0.4	0.0005 – 1.000	0.011 ± 0.0	3.05E-05 – 0.0625	-	-
CEM/BTZ200	2.261 ± 1.1	0.008 – 16.000	0.011 ± 0.0	3.05E-05 – 0.0625	-	-
AML cells						
THP-1/WT	0.018 ± 0.0	3.05E-05 – 0.0625	-	-	1.457 ± 0.5	0.005 – 10.000
THP-1/BTZ7	0.538 ± 0.1	0.0005 – 1.000	-	-	0.945 ± 0.3	0.005 – 10.000
THP-1/BTZ200	4.787 ± 1.2	0.008 – 16.000	-	-	0.380 ± 0.1	0.001 – 2.500

Results are expressed as cell growth relative to control cells incubated without drug, set at 100%. Mean ± SD (in μM) of three separate experiments performed in triplicate. Concentration ranges depict the lowest and highest concentrations of total 12 concentrations (μM)
